# Platinum nanosheets synthesized via topotactic reduction of single-layer platinum oxide nanosheets for electrocatalysis

**DOI:** 10.1038/s41467-022-35616-4

**Published:** 2023-01-09

**Authors:** Daisuke Takimoto, Shino Toma, Yuya Suda, Tomoki Shirokura, Yuki Tokura, Katsutoshi Fukuda, Masashi Matsumoto, Hideto Imai, Wataru Sugimoto

**Affiliations:** 1 Research Initiative for Supra-Materials (RISM), Shinshu University, 3-15-1 Tokida, Ueda, Nagano, 386-8567 Japan; 2 Faculty of Science, University of the Ryukyus, 1-Senbaru, Nishihara, Nakagami, Okinawa, 903-0213 Japan; 3 Faculty of Textile Science and Technology, Shinshu University, 3-15-1 Tokida, Ueda, Nagano, 386-8567 Japan; 4 Office of Society-Academia Collaboration for Innovation, Kyoto University, Sakyo-ku, Kyoto, 606-8501 Japan; 5 Device-functional Analysis Department, NISSAN ARC LTD., 1 Natsushima, Yokosuka, Kanagawa 237-0061 Japan

**Keywords:** Electrocatalysis, Electrocatalysis, Fuel cells, Two-dimensional materials

## Abstract

Increasing the performance of Pt-based electrocatalysts for the oxygen reduction reaction (ORR) is essential for the widespread commercialization of polymer electrolyte fuel cells. Here we show the synthesis of double-layer Pt nanosheets with a thickness of 0.5 nm via the topotactic reduction of 0.9 nm-thick single-layer PtO_*x*_ nanosheets, which are exfoliated from a layered platinic acid (H_*y*_PtO_*x*_). The ORR activity of the Pt nanosheets is two times greater than that of conventionally used state-of-the-art 3 nm-sized Pt nanoparticles, which is attributed to their large electrochemically active surface area (124 m^2^ g^−1^). These Pt nanosheets show excellent potential in reducing the amount of Pt used by enhancing its ORR activity. Our results unveil strategies for designing advanced catalysts that are considerably superior to traditional nanoparticle systems, allowing Pt catalysts to operate at their full potential in areas such as fuel cells, rechargeable metal–air batteries, and fine chemical production.

## Abstract (web summary)

2D metals are promising electrocatalysts due to their potentially large surface area. Here we report double-layer Pt nanosheets derived from exfoliated PtOx nanosheets with higher electrochemically active surface area, oxygen reduction reaction activity, and stability compared to Pt nanoparticles.

## Introduction

Limiting the usage of Pt by increasing the performance of Pt-based electrocatalysts for the oxygen reduction reaction (ORR) is essential for the widespread commercialization of polymer electrolyte fuel cells^1–4^. Pt-based electrocatalysts, such as alloys and core–shell structures, have been reported to show high ORR mass activity and thus are potential candidates for cathodes in polymer electrolyte fuel cells^5–8^. However, complications arise when such catalysts are exposed to the harsh operating conditions of fuel cells, which cause Pt to undergo consecutive oxidation/reduction and dissolution, resulting in the second metal becoming prone to dissolution and deactivation of the catalyst^9–11^. Additionally, the use of Pt-group metals, such as Au and Pd, must be minimized to improve the overall cost performance of these catalysts.

Nanoparticle systems have certain disadvantages in terms of their ORR performance. While the electrochemically active surface area (ECSA) increases with decreasing particle size, the ORR specific activity gradually decreases with decreasing particle size. Since the ORR mass activity is the product of ECSA and the ORR specific activity, the mass activity of Pt shows Sabatier-volcano-type plot characteristics as a function of nanoparticle size. Therefore, a trade-off relationship exists between the ECSA and the specific activity for Pt nanoparticle (Pt NP) systems in terms of Pt utilization. A maxima in the ORR mass activity is achieved for 3 nm-sized Pt NPs^12–14^, and based on a spherical particle model, the Pt efficiency of 3 nm-sized Pt NPs is estimated to be 40% (60% is not used in the reaction). Thus, enhancement in Pt efficiency is desired. However, smaller nanoparticles with high ECSA (higher Pt utilization) have low ORR specific activity. In addition, as cluster-sized Pt NPs generally have a higher rate of dissolution and growth rate^15–19^ and thus insufficient durability, they are not suitable for practical fuel cells. Therefore, a nanostructure that can afford high ECSA of NPs with ORR specific activity and stability of bulk Pt would be an ideal Pt electrocatalyst.

**Fig. 1:**
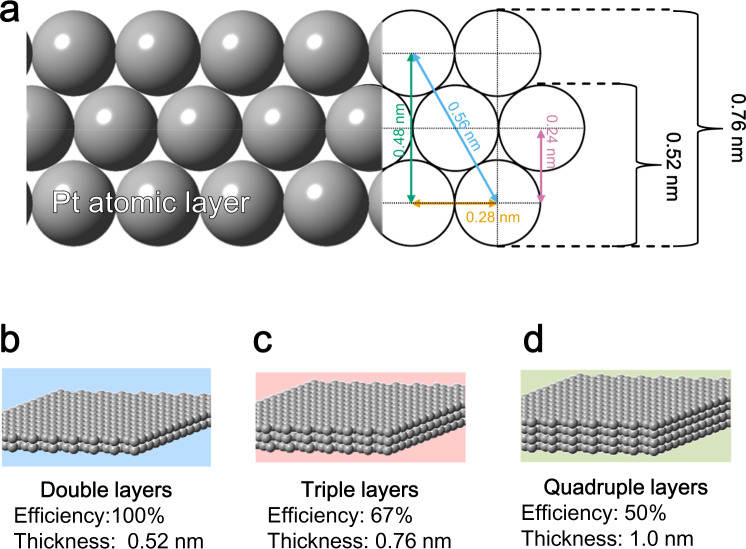
Efficiency and thickness of Pt nanosheet (NS). **a** Cross-sectional view of Pt NS and calculation thickness. Schematic images of (**b**) double-, (**c**) triple-, and **d** quadruple-layer Pt NSs.

Two-dimensional (2D) materials, such as nanosheets, have attracted considerable attention owing to their large surface area^20–28^. In principle, metal nanosheets with single- or double-atomic layer(s) (ALs) can utilize 100% of their constituent atoms (Fig. 1); therefore, the resulting high electrochemical activity is expected to facilitate their applications as electrocatalysts. Assuming a Pt–Pt distance of 0.28 nm, the thicknesses of a Pt nanosheet (NS) with 1–4 ALs are calculated to be 0.28, 0.52, 0.76, and 1.0 nm, respectively (Fig. 1b–d). Thus, a 4 AL-Pt NS with a thickness of ~1.0 nm corresponds to a Pt efficiency of 50%, which is still slightly higher than that of the 3 nm-sized standard-performance Pt NPs.

We recently reported that metal nanosheets are more electrocatalytically active and stable than their nanoparticle equivalents^29^. Consequently, 2D Pt can be a potential candidate for a fuel cell electrocatalyst. However, only a few studies have reported on the synthesis of 2D Pt; for example, few-nanometer-thick Pt nanoplates have been prepared in the interlayers of graphene oxide^30,31^. A combination of exfoliation and topotactic reduction was used to fabricate 1 nm-thick Pt nanoplates^32^. However, the thickness of these Pt NSs is still more than 1 nm, leading to low efficiency and forming materials that do not effectively reflect the 2D nature. The thick Pt nanoplates could be attributed to the fairly thick (thickness of 1.5 nm) structure of the exfoliated PtO_2_ NSs^32^. Thus, synthesizing atomically thin Pt NSs with thicknesses of less than 1 nm (<4 ALs) is desirable and challenging. One of the challenges associated with obtaining atomically thin Pt NSs is the limited control of the nanosheet thickness during synthesis. We previously synthesized 2 AL-Ru NSs via the topochemical reduction of single-layer RuO_2_ NSs, which were obtained by exfoliating the layered RuO_2_^33^. Although the topotactic reduction of oxide nanosheets is a promising approach for obtaining atomically thin metal nanosheets, the thickness of the oxide nanosheets should preferably be a single layer. Therefore, the synthesis of single-layer Pt oxide NSs is required to obtain atomically thin Pt NSs.

**Fig. 2:**
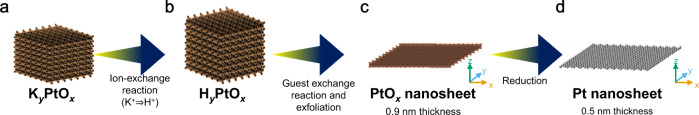
Synthetic approach of double-layer Pt NSs. **a** Layered K_*y*_PtO_*x*_ was synthesized by the solid-state-reaction of a mixture of K_2_CO_3_ and PtO_2_. **b** The acid-treated form, layered H_*y*_PtO_*x*_, was prepared via the ion-exchange reaction. **c** PtO_*x*_ NS was obtained via exfoliation of layered H_*y*_PtO_*x*_. **d** Pt NS was synthesized via topotactic reduction of PtO_*x*_ NS.

Herein, we report the synthesis of 0.5 nm-thick 2 AL-Pt NSs via the topotactic reduction of 0.9 nm-thick PtO_*x*_ NSs that were exfoliated from a layered potassium platinate (Fig. 2). The 2 AL-Pt NSs outperformed commercial 3 nm-sized Pt NPs in terms of ECSA, ORR mass activity, and durability against potential cycling, finally breaking the trade-off relationship in nanoparticle catalysts. The developed atomically thin Pt NS may pave a path for 2D catalysts with excellent activity and durability for energy conversion and storage and other practical applications where Pt NPs are state-of-the-art.

## Results

### Preparation and characterization

**Fig. 3:**
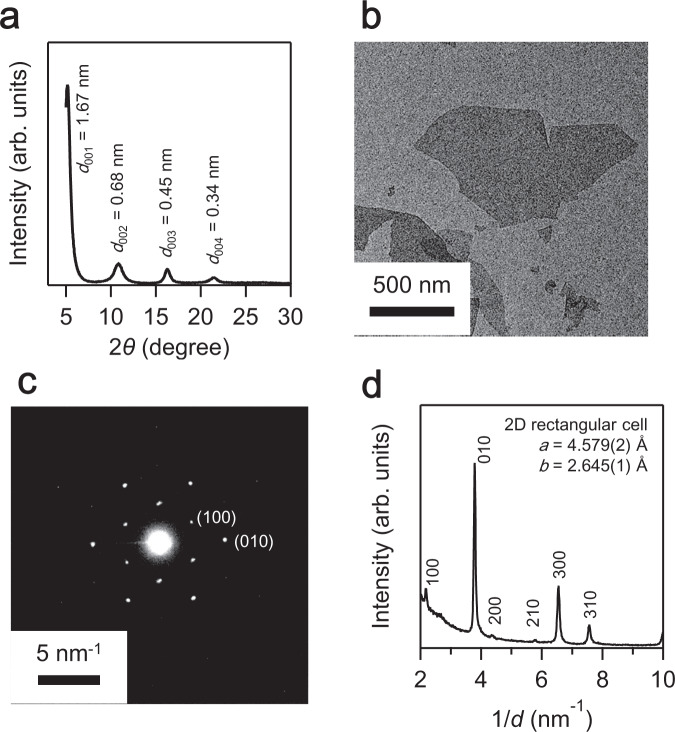
Characterization of the PtO_*x*_ NSs. **a** X-ray diffraction (XRD) pattern of a film cast from the PtO_2_ NS colloid. **b** A typical transmission electron microscopy (TEM) image of a PtO_*x*_ NS and the corresponding **c** selected-area electron diffraction pattern. **d** In-plane XRD pattern of a monolayer PtO_*x*_ NS film on a Si substrate.

A yellow PtO_*x*_ NS colloid was obtained by exfoliating the layered platinic acid (H_*y*_PtO_*x*_) in an aqueous solution containing tertbutylammonium ion (TBA^+^). The solution remains stable without any sedimentation, and the observation of Tyndall phenomena with a laser beam (Supplementary Fig. 1) confirms its colloidal nature. H_*y*_PtO_*x*_ was derived from a solid-state reaction between K_2_CO_3_ and PtO_2_ and the subsequent acid treatment (Supplementary Figs. 2 and 3). The X-ray diffraction (XRD) pattern of a thin film prepared by casting the PtO_*x*_ NS colloid onto a glass plate exhibited a series of diffraction peaks with *d* = 1.67, 0.83, 0.55, and 0.42 nm, which were indexed as high-order diffraction peaks with a basal spacing of 1.67 nm (Fig. 3a). The results show that casting the colloid led to the flocculation of negatively charged PtO_*x*_ NS with the counter cation TBA^+^ to form a TBA^+^–PtO_*x*_ intercalation compound. Based on the basal spacing and molecular size (0.8–0.9 nm)^34^ of the guest species TBA^+^, the crystallographic thickness of PtO_*x*_ NSs was estimated to be ~0.8 nm.

A typical transmission electron microscopy (TEM) image of the PtO_*x*_ NSs revealed micrometer-sized nanosheets with low contrast, typical of a thin morphology (Fig. 3b). The selected-area electron diffraction (SAED) pattern showed sharp spots typical of single crystals, which were indexed based on a hexagonal cell with lattice parameters *a* = 4.57 Å and *b* = 2.64 Å (Fig. 3c). Figure 3d shows the in-plane XRD pattern for a PtO_*x*_ NS monolayer formed on Si (PtO_*x*_-NS/Si) by electrostatic interactions. All the peaks were indexed to a 2D rectangular primitive cell with *a* = 4.579(2) Å and *b* = 2.645(1) Å, consistent with the cell parameters obtained from SAED analysis.

**Fig. 4:**
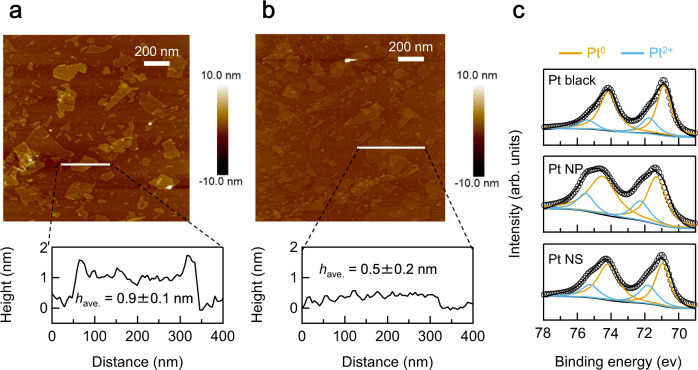
Thickness and electronic states of the Pt NSs. Typical atomic force microscopy images and the corresponding height profiles of (**a**) PtO_*x*_-NS/Si and (**b**) Pt-NS/Si films. **c** Pt 4 f XPS profiles of Pt black, 3 nm-sized Pt NPs, and Pt NSs.

Atomic force microscopy (AFM) images of PtO_*x*_ NSs and their corresponding height profiles indicated that their average thickness was 0.9 ± 0.1 nm (Fig. 4a and Supplementary Fig. 4). The thickness is consistent with the crystallographic thickness of ~0.8 nm estimated from XRD (Fig. 3a). The sample was subjected to H_2_ gas at various temperatures to reduce PtO_*x*_ NS/Si to Pt NS/Si. The in-plane XRD pattern of a sample reduced at 100 °C was indexed as face-centered cubic Pt (Supplementary Fig. 5). After the reduction, the average thickness of the nanosheets decreased to 0.5 ± 0.2 nm, which was considerably lower than that of the PtO_*x*_ NSs (Fig. 4b and Supplementary Fig. 4). Based on the anticipated geometry for Pt NSs shown in Fig. 1, the Pt NSs deposited on the Si wafer should comprise 2 AL-Pt NS at the most. All of the diffraction peaks in the in-plane XRD pattern of the reduced sample were indexed to a cubic lattice with a cell parameter *a* = 3.920(1) Å (Supplementary Fig. 5), consistent with bulk Pt (3.92 Å).

The electronic states of the Pt NSs were characterized by core-level X-ray photoelectron spectroscopy (XPS). The binding energies of the Pt 4f_5/2_ and Pt 4f_7/2_ peaks for Pt NSs were detected at 74.3 and 71.1 eV, respectively (Fig. 4c). The peaks associated with Pt^4+^ (78.2 eV for Pt 4f_5/2_ and 74.9 eV for Pt 4f_7/2_) disappeared after the H_2_ treatment, indicating successful reduction (Supplementary Fig. 6). The spectra for the reduced sample were deconvoluted into two components: Pt^0^ and Pt^2+^. Pt^2+^ was also observed for the 3 nm-sized Pt NPs and Pt black, which is attributed to surface oxidation due to atmospheric exposure. The content of Pt^0^ in 0.5 nm-thick Pt NSs was 78%, which was between the content for ~20 nm-sized Pt black (82%) and 3 nm-sized Pt NPs (73%).

The structural and chemical characterization is sound evidence confirming the successful synthesis of a colloidal solution containing 0.9 nm-thick single-crystalline PtO_*x*_ NSs with a lateral size of a few hundred nanometers and the topotactic metallization to 0.5 nm-thick Pt NSs by a mild thermal treatment in H_2_ (Fig. 2).

### Characterization of Pt NSs supported on carbon

The thicknesses of metal nanosheets are affected by the morphology and roughness of the support material^35^. The thickness of the Pt NSs supported on carbon could not be directly determined by AFM and TEM. Hence, in situ X-ray absorption spectroscopy (XAS) measurements were performed to gain information on the state of Pt NSs. The coordination number (CN) was targeted as a parameter of interest because it could, in principle, help determine the thickness of metal nanosheets; the CN values for an ideal nanosheet with infinite size would be CN = 6, 9, and 10 for 1, 2, and 3 AL-nanosheet, respectively. The average CN (CN_ave._) of the Pt NSs obtained by multiple-shell fitting in *r*-space was 10.6 ± 0.4 (Supplementary Figs. 7–9), indicating that the Pt NSs were predominantly 3 AL-Pt NSs. The difference between the thickness of the Pt NSs on the Si wafer (2 ALs) and those on a carbon support (3 ALs) may have been affected by the roughness of the carbon support; the use of a non-flat porous carbon may have resulted in an uneven reduction process.

### Electrocatalytic performance

**Fig. 5:**
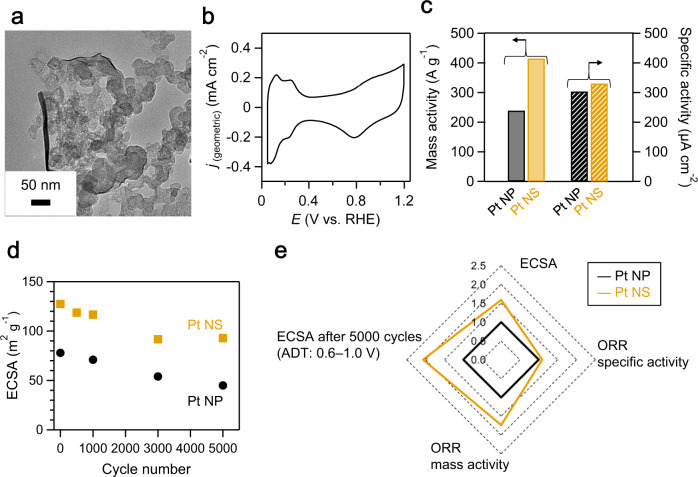
Electrochemical performance of Pt NSs. **a** Typical TEM image of Pt NSs supported on carbon (EC-300J). **b** Cyclic voltammogram of Pt NSs collected using de-aerated 0.1 M HClO_4_ (25 °C) at a scan rate of 50 mV s^−1^. **c** Mass and specific activity of fresh samples for the oxygen reduction reaction (ORR) at 0.9 V vs. reversible hydrogen electrode (RHE). **d** Electrochemically active surface area (ECSA) as a function of potential cycle (load-cycle accelerated durability test; 0.6–1.0 V vs. RHE at 60 °C). **e** Comparison of electrocatalytic performance of Pt NSs and 3 nm-sized Pt NPs. The values are normalized by those of the 3 nm-sized Pt NPs.

The electrocatalytic performance of the Pt NSs was investigated to clarify their structural characteristics by comparing them with conventional 3 nm-sized Pt NPs (Supplementary Fig. 10). Figure 5a illustrates a typical TEM image, showing a Pt NS covering the carbon support maintaining its nanosheet morphology. The cyclic voltammogram in de-aerated 0.1 M HClO_4_ showed typical features of polycrystalline Pt with hydrogen adsorption/desorption peaks below 0.4 V and surface oxidation/reduction at high potentials (Fig. 5b). The ECSA was characterized by the charge associated with hydrogen adsorption in the cathodic branch below 0.4 V in cyclic voltammograms. The ECSA of the Pt NSs was 124 m^2^ (g-Pt)^−1^, which was greater than that of the 3 nm-sized Pt NPs (79 m^2^ (g-Pt)^−1^; Supplementary Tables). The high ECSA was also confirmed by CO-stripping voltammetry (Supplementary Fig. 11). This result indicates that the large ECSA of the Pt NSs could be attributed to their ultrathin nature.

The ORR activity was investigated in 0.1 M HClO_4_ using a rotating disk electrode. The Koutecký–Levich equation was used to calculate the kinetic current at 0.9 V accumulated from linear sweep voltammetry at different rotation rates. In addition to the high ECSA, the Pt NSs showed a high mass activity (415 A g^−1^) that was nearly double that of the Pt NPs (Fig. 5c, Supplementary Tables, and Supplementary Fig. 12).

In addition to their high ORR mass activity, the Pt NSs exhibited high durability with respect to the dissolution of Pt (Fig. 5d and Supplementary Figs. 12–15). The ECSA of the Pt NSs subjected to 5000 cycles decreased from 124 to 93 m^2^ g^−1^ (75% retention), which was two times higher than that of the Pt NPs (45 m^2^ g^−1^ after cycling, 57% retention). The ultrathin Pt NSs outperformed the conventional 3 nm Pt NPs in terms of ECSA, mass activity, and durability (Fig. 5e). The morphology of the Pt NSs remained unchanged or marginally aggregated after the durability test (Supplementary Fig. 15). In contrast, severe aggregation and increased particle size were observed after the durability test for Pt NPs (Supplementary Fig. 15). These results indicate that Pt NSs are more stable than Pt NPs in terms of morphological changes (dissolution/re-deposition of Pt atoms).

## Discussion

**Fig. 6:**
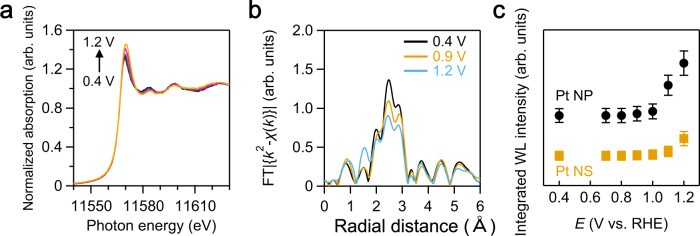
Characterization of surface oxidation state of Pt NSs. **a** X-ray absorption near-edge structure spectra of the Pt NSs at various potentials (0.4, 0.7, 0.8, 0.9, 1.0, and 1.1 V). **b** Fourier transform of the *k*^2^-weighted extended X-ray absorption fine structure data of the Pt NSs at various potentials. **c** Ratio of the white-line (WL) intensity to Pt surface area of the Pt NPs and Pt NSs as a function of electrode potential.

Two key points are notable regarding the electrocatalytic properties of Pt NSs: high ORR specific activity and durability. First, we discuss from the perspective of the ORR specific activity of Pt NSs. Despite the higher ECSA, the ORR specific activity of the Pt NSs was comparable with that of the Pt NPs. Thus, the so-called particle-size effect and trade-off between the ECSA and specific activity were circumvented by changing the morphology from nanoparticle to nanosheet. The high specific activity of Pt electrocatalysts is attributed to the suitable adsorption energy of oxygen species on the Pt surface. Strong adsorption of oxygen species on the Pt during ORR leads to the blocking of the ORR sites, while the reaction is suppressed due to the lack of chemical bonding when the adsorption is very weak^9^. This aspect can be accurately characterized by the extent of surface-covered oxygen determined by in situ XAS at a given potential with respect to the electrical charge associated with the surface oxidation reaction of Pt^36^. Thus, oxygen coverage was evaluated as a function of electrode potential by in situ XAFS to investigate the high specific activity of the Pt NSs for the ORR. The X-ray absorption near-edge structure (XANES) spectra and *k*^2^-weighted Fourier-transform extended X-ray absorption fine structure (FT-EXAFS) oscillation spectra of the Pt NSs showed behavior similar to that of the Pt foil and Pt NPs (Supplementary Figs. 7–9). These results indicate that the Pt NSs were completely reduced to Pt. The XANES spectra obtained at a fixed potential at the Pt *L*_III_ adsorption edge (Fig. 6a) were indexed to Pt, and an evident increase in the white-line (WL) intensity was observed, along with a shift to higher energies. This was attributed to an increase in the unoccupied Pt 5*d* states due to the charge transfer from Pt to adsorbed oxygen species^36^. The radial distribution function was comprehensively analyzed to obtain greater insight into the structures of the surface oxides. In the FT-EXAFS spectra of the Pt foil, 3 nm-sized Pt NPs, and Pt NSs (Fig. 6b), the FT amplitude at ~2.7 Å (corresponding to the first nearest-neighbor Pt–Pt bond) decreased with increasing electrode potential, whereas that corresponding to radial distances of less than 2 Å (two types of Pt–O bonds) increased with increasing electrode potential. These results indicate the formation of surface oxide layers on the Pt NSs. The degree of surface oxidation of the nanoparticles and nanosheets was compared by monitoring the WL amplitude as a function of electrode potential. The integrated WL intensity was normalized to the exposed Pt surface area (Fig. 6c). The surface oxidation of the Pt NSs was significantly suppressed compared to that of the Pt NPs. This phenomenon is attributed to the difference in the adsorption energy of oxygen species, which typically blocks the active sites intended for the ORR^3^.

Next, we discuss the high stability of the Pt NSs. The suppression of the surface oxidation of Pt catalysts is widely known to enhance catalytic stability^37^. This could be attributed to the suppression of the dissolution of Pt ions during the potential cycles^11^. The dissolution of Pt ions is accelerated at a low CN_ave._ of the Pt atoms^38^, leading to the growth of Pt nanoparticles. In addition, improved stability of Pt catalysts has been reported for morphology-controlled catalysts^39,40^. Although most studies did not discuss the mechanism of the improved stability, we have recently reported the mechanism of the high oxidation tolerance of Ru NSs^29^. Based on the combination of in situ XAS measurements and XANES spectral simulations, the CN_ave._ of the Ru atoms on the surface of the nanosheet was found to be larger than that on the surface of the nanoparticle; thus, the Ru NSs showed higher oxidation tolerance. The CN_ave._ of the Pt atoms for Pt NPs was 10.6 ± 2.8 (Supplementary Figs. 7–9), while the CN_ave._ of the surface was 6–9^41^, indicating that the surface of the Pt NPs analyzed in this study was composed of Pt with a low CN. In contrast, considering that most of the Pt atoms for the 3 AL-Pt NSs are on the surface, the CN_ave._ of the Pt NSs (10.6 ± 0.4) was higher than that of Pt NPs. This suggests that the Pt atoms of the Pt NSs are more stable than those of the Pt NPs with respect to the surface oxidation, which accelerates the dissolution of Pt atoms. Hence, the electrochemical stability of the Pt NSs was higher than that of the Pt NPs in this study. Based on this background, the selection of Pt NSs is an alternative for obtaining ECSA, specific activity, and durability higher than those of the Pt NPs. While the present study focuses on the synthesis of atomically thin Pt NSs and their electrocatalytic performance, the mechanistic understanding of the enhanced stability of Pt NSs is undoubtedly an important aspect of this study. According to a study on the dissolution behavior of Pt, the dissolution of Pt is intricately related to the oxide structure on the Pt surface, electronic state, and CN^11^. Because these properties strongly depend on the nanostructure, Pt NSs may exhibit different dissolution behavior than nanoparticles. For a complete understanding of the electrochemical stability of Pt NSs, a combination of more comprehensive and sophisticated approaches such as inductively coupled plasma-mass spectrometry (ICP-MS), in situ XAS, and in situ scanning tunneling microscopy measurements is beneficial and presently under consideration.

In summary, this study demonstrates the successful synthesis of Pt NSs with atomic-level thickness and their application toward highly active and durable ORR electrocatalysts. The 0.5 nm-thick, 2 AL-Pt NSs were prepared via topotactic metallization of single-layer PtO_*x*_ NSs. The topotactic conversion of PtO_*x*_ to Pt NS was confirmed by various analytical methods, including microscopy, diffractometry, and spectroscopy (such as TEM, AFM, in-plane XRD, XPS, and XAS). The ECSA of the Pt NSs was 124 m^2^ (g-Pt)^−1^, 1.6 times greater than that of 3 nm-sized Pt NPs, which is a conventional electrocatalyst. Owing to their large ECSA, the Pt NSs exhibited a mass activity for the ORR, which was 1.7 times higher than that of the 3 nm-sized Pt NPs. Despite the higher ECSA, the Pt NSs showed higher durability than the Pt NPs, which is unusual for the nanoparticle systems. Interestingly, the ORR specific activity of the Pt NSs was comparable with that of the Pt NPs, despite the large ECSA. This result indicates that the Pt NSs overcame the trade-off between ECSA versus durability and the specific activity for the ORR (that is, particle-size effects). In situ XAFS aided in attributing the high ORR specific activity to the suppressed formation of surface oxygen layers on the Pt NSs during the ORR. These findings can open fields and opportunities for employing Pt NSs in electrochemical energy conversion and storage, as well as numerous other applications where Pt NPs are presently used.

## Methods

### Preparation of layered potassium platinate and exfoliated platinum-oxide nanosheets

The solid-state-reaction conditions were optimized by varying the starting material, temperature, and reaction time. Typical synthetic conditions are summarized herein. A 1:1 mixture of K_2_CO_3_ and PtO_2_ (FUJIFILM Wako Pure Chemical Industries) was pelletized and calcined at 800 °C for 1 h in air. The pellet was pulverized in an inert atmosphere, pelletized again, and calcined at 800 °C for 1 h in air; the heat treatment was performed twice. The final product was washed with Milli-Q ultrapure water (>18 MΩ·cm, Millipore) to remove water-soluble impurities. The product was treated with 1 M HCl (FUJIFILM Wako Pure Chemical Industries) for three days, and the acidic solution was replaced daily. The acid-treated sample was reacted with an aqueous solution of 10% tetrabutylammonium hydroxide (TBA^+^OH^‒^; FUJIFILM Wako Pure Chemical Industries) for one week. Subsequently, the suspension was centrifuged at 4000 rpm (2600 G) for 30 min to remove the unexfoliated material, which resulted in a colloid containing PtO_*x*_ NSs (~0.3 g L^−1^). The Pt content in the colloid was determined by ICP-MS (Agilent 7700, Agilent Technologies). The colloid (1 mL) was added to concentrated HCl (9 mL; FUJIFILM Wako Pure Chemical Industries) in a 23 mL polytetrafluoroethylene cup liner, which was closed and slid into a temperature- and pressure-tolerant metal vessel (4749, Parr Instrument Company) and heated at 180 °C for 2 h. Prior to the ICP-MS-based determination, the solution was further diluted with ultrapure water, and the concentration of HCl solvent was adjusted to 1 M.

### Characterization of PtO_*x*_ NSs

The structure of the layered K_*y*_PtO_*x*_ was confirmed by powder XRD analysis, which generated diffraction peaks at *d* = 0.68 and 0.34 nm (Supplementary Fig. 2). These peaks could not be indexed to known phases composed of K, Pt, and O. The diffraction peaks of this unidentified phase shifted to smaller angles at *d* = 0.72 and 0.36 nm after acid treatment. Field-emission scanning electron microscopy revealed micron-sized particles with at least two types of morphology (platelet-like and square-shaped crystallites) (Supplementary Fig. 3). Numerous cracks on the platelets suggested a layered-type structure, whereas the platelet-like crystallites, which are not typical of PtO_2_ or Pt, were presumably the unidentified phase identified by XRD (*d* = 0.72 nm and 0.36 nm). After the acid treatment, copious cracks were observed in the platelets, suggesting an opening up of the framework and a reactive interlayer (Supplementary Fig. 3). X-ray photoelectron spectroscopy analysis indicated that K was not detected after the acid treatment. These observations are typical of the interlayer expansion that occurs upon proton exchange in ion-exchangeable layered oxides and are consistent with the observed changes in XRD patterns, morphology, and cation ratio due to the acid treatment. These results indicate that the acid treatment of K_*y*_PtO_*x*_ with a basal spacing of 0.68 nm yielded H_*y*_PtO_*x*_ with a basal spacing of 0.72 nm via a proton-exchange reaction (Supplementary Fig. 2). The obtained H_*y*_PtO_*x*_ was added to a tetrabutylammonium hydroxide solution and vigorously shaken for 7 days. Yellow suspensions that exhibited Tyndall scattering with a laser beam were obtained (Supplementary Fig. 1), indicating their colloidal nature. The XRD pattern of a thin film prepared by casting the PtO_*x*_ NS colloid onto a glass plate exhibited a series of diffraction peaks, which were indexed as high-order diffraction peaks with a basal spacing of 1.67 nm (Fig. 3a).

### Optimization of the conditions for Pt NS synthesis

The in-plane XRD patterns of PtO_*x*_-NS/Si indicated that the PtO_*x*_ NSs were transformed into Pt NSs via heat treatment at temperatures above 100 °C but remained slightly in their oxide form at 50 °C (Supplementary Fig. 5). Although the two-dimensional morphology was retained at 100 °C, the orientation of the Pt NSs was lost at temperatures above 200 °C. The thicknesses of the PtO_*x*_ and Pt NSs were estimated to be 0.9 and 0.5 nm, respectively.

### Synthesis of carbon-supported Pt NS catalyst

An aqueous suspension of carbon (Ketjen black; EC-300J, Lion Corp.) was added to the PtO_*x*_ NS colloid. The dispersion was magnetically stirred (~500 rpm) and ultrasonicated (30 min) to ensure a homogeneous dispersion. After sedimentation, the composite was washed with ultrapure water and dried to yield carbon-supported PtO_*x*_ NSs in powder form (PtO_*x*_-NS/C). The PtO_*x*_-NS/C system was then subjected to a constant flow of H_2_ gas at 100 °C for 2 h to prepare carbon-supported Pt-NS/C (12.6 mass% Pt). The Pt content in the catalysts was estimated by ICP analysis (ICPE-9800, Shimadzu). To this end, the powder (~1 mg) was dispersed in aqua regia (4 mL), and the dispersion was transferred to the previously mentioned Teflon-lined stainless-steel autoclave and heated at 200 °C for 12 h. The solution was subsequently diluted to 25 mL with ultrapure water, and the resulting specimen was analyzed.

### Morphological and structural analysis

The morphologies of the electrocatalysts were examined by TEM (JEOL 2010, JEOL; accelerating voltage, 200 kV). The height and morphology of the PtO_*x*_ NSs deposited on Si wafers were determined by tapping-mode AFM (Digital Instruments Nanoscope IIIa, Bruker). This analysis was facilitated by immersing the wafer for 10 s in an aqueous solution of poly(diallyldimethylammonium chloride) (2 wt.%), which was used as the counter polycation. Subsequently, the wafer was dipped into a colloidal suspension of negatively charged PtO_*x*_ NSs for 30 s to assemble a monolayer film. The PtO_*x*_-NS/Si film was placed in a tubular furnace and reduced under hydrogen flow at 100 °C for 2 h. AFM images were acquired in the tapping mode using a Si-tip cantilever with force constant of 40 N m^−1^ and resonance frequency of 320 kHz under atmospheric conditions (NCHV-A, Bruker). The crystal structure was characterized by XRD (Rigaku MiniFlex 600 with monochromatic Cu Kα radiation at 40 kV and 15 mA). XPS (AXIS-ULTRA DLD, Kratos Analytical; 15 kV and 10 mA, Mg Kα standard) was performed to analyze the chemical states of Pt. The sample was loaded on the carbon tape, followed by the addition of Au powder. The binding energies were charge-corrected to that of Au 4 f, and the measurements were conducted without Ar^+^ sputtering. In-plane XRD data of a PtO_*x*_ NS monolayer film prepared via electrostatic self-assembly on a Si substrate were acquired using a four-axis diffractometer equipped with a NaI scintillation counter at the BL-6C beamline of the Photon Factory at the High Energy Accelerator Research Organization.

### Cathodic activity and durability measurements

Electrochemical measurements were conducted using a rotating disk electrode (SC-5, Nikko Keisoku) connected to an automatic polarization system (HSV-100, Hokuto Denko). Carbon fiber (HTA40 E13 3 K 200tex, Toho Tenax) and a reversible hydrogen electrode (RHE) were used as the counter and reference electrodes, respectively. The electrochemical properties of the Pt NSs were compared with those of commercial carbon-supported 3 nm-sized Pt NPs (46.3 mass% Pt; TEC10E50E, Tanaka Kikinzoku Kogyo K.K.), which was typically used as standard-performance catalysts in various studies^42,43^. Typical TEM images and the corresponding particle size distributions of these particles are shown in Supplementary Fig. 10. The catalyst ink was prepared by dispersing the catalyst in a 2-propanol/water solution (75/25 vol.%; 18.5 mg/25 mL and 2.5 mg/5 mL for Pt NPs and Pt NSs, respectively) and Nafion® solution (5 wt.%; Sigma-Aldrich; 100 and 20 μL for Pt NPs and Pt NSs, respectively). The working electrode was prepared by dropping the catalyst ink onto a mirror-polished 6 mm-diameter glassy carbon rod. The electrocatalyst was loaded onto the glassy carbon electrode at Pt mass loadings of 17.3 and 2.36 µg cm^−2^ for the 3 nm-sized Pt NPs and Pt NSs, respectively. The electrolyte was de-aerated with N_2_ gas unless otherwise noted. Prior to the electrochemical measurements, the surface of the catalyst was electrochemically cleaned by cycling in 0.1 M HClO_4_ (25 °C; Tamapure-AA-100, Tama Chemicals) between 0.05 and 1.2 V vs. RHE at a scan rate of 50 mV s^−1^. Cyclic voltammetry was conducted in de-aerated 0.1 M HClO_4_ at a scan rate of 50 mV s^‒1^ (*E* = 0.05 and 0.9 V at 25 °C), and ECSAs were estimated using the hydrogen adsorption (H_UPD_) charge determined by excluding the hydrogen evolution area and the Coulombic charge required for oxidation on polycrystalline Pt (210 µC cm^−2^)^44^. Pre-adsorbed CO (CO_ad_) stripping voltammetry was conducted to estimate the CO oxidation activity and ECSA of the catalysts. Gaseous CO was purged into the electrolyte for 40 min while maintaining a constant potential of 100 mV vs. RHE. Excess CO in the electrolyte was purged out by bubbling N_2_ gas for 40 min. CO_ad_ was electro-oxidized by voltammetry at a scan rate of 10 mV s^‒1^. The amount of CO_ad_ was estimated by integration of the CO_ad_ stripping peak and corrected for the electrical double-layer capacitance, assuming a monolayer of linearly adsorbed CO on the metal surface and the Coulombic charge necessary for oxidation to be 420 μC cm^‒2^. Linear sweep voltammetry was conducted in O_2_-saturated 0.1 M HClO_4_ (25 °C) at rotation rates (ω) of 2500, 2200, 1600, 1200, 800, and 400 rpm. Background subtraction was performed by subtracting the linear sweep voltammograms collected under de-aerated conditions (N_2_ gas) at the respective rotation rates from those obtained using the O_2_-saturated electrolyte. The potential was swept from 0.05 to 1.2 V vs. RHE at 10 mV s^−1^. The ORR activity was obtained by extrapolating the Koutecký–Levich plots obtained at 0.9 V vs. RHE to ω = ∞, which yielded the kinetically controlled current (no *iR* correction). The ORR mass activity of the catalyst was determined as the ratio of the kinetically controlled current at 0.9 V vs. RHE to the mass of Pt on the working electrode. The ORR specific activity was estimated as the ratio of the kinetically controlled current at 0.9 V vs. RHE to the ECSA of the catalyst measured based on the H_UPD_ region. The load-cycle accelerated durability test was conducted by potential stepping between 0.6 and 1.0 V vs. RHE (a 3 s hold for each potential) at 60 °C for 5000 cycles (ω = 0).

### XAS

The in situ Pt *L*_III_-edge XAS measurement (fluorescence mode, Si(311) double-crystal monochromator) was performed at the BL14B2 and BL36XU beamlines of SPring-8 (Hyogo, Japan) to obtain the structural and electronic structural information of the Pt NSs and NPs. The catalyst ink was dropped on a carbon plate substrate and subsequently dried at 80 °C (50 μg-Pt on substrate). The sample was immersed in a methanol/water solution to improve the wettability of the hydrophobic catalyst layer. A polychlorotrifluoroethylene-based in situ electrochemical cell was used for the in situ XAS analysis, as described in previous studies^29,45^. Pt wire and a RHE were used as the counter and reference electrodes, respectively. The surface oxygen species were removed by potential cycles at 100 mV s^−1^ (0.05 − 0.8 V vs. RHE for Pt NSs and 0.05–1.2 V vs. RHE for Pt NPs) in de-aerated 0.1 M HClO_4_. The spectra were recorded at each potential after a certain number of cleaning cycles; typically, 5 cycles for Pt NSs and 20 to 30 cycles for Pt NPs. The data were analyzed using the Athena and Artemis programs, and an *R*-space fitting was performed with multiple *k*-weights (1, 2, 3).

## Supplementary information

Supplementary Information

## Data Availability

The authors declare that all other data supporting the findings of this study are available within the article and its supplementary information files.
