# Supplementary material for: Platinum nanosheets synthesized via topotactic reduction of single-layer platinum oxide nanosheets for electrocatalysis
Source: Nat Commun. 2023 Jan 9;14:19. doi: 10.1038/s41467-022-35616-4 (PMC9829898; doi:10.1038/s41467-022-35616-4)
Supplement: Supplementary file 1 — Supplementary Information [file 41467_2022_35616_MOESM1_ESM.pdf]

Supplementary Information

Platinum nanosheets synthesized via topotactic reduction of single-layer platinum oxide nanosheets for electrocatalysis

Daisuke Takimoto[1,2,*], Shino Toma[2], Yuya Suda[3], Tomoki Shirokura[3], Yuki Tokura[3], Katsutoshi Fukuda[4], Masashi Matsumoto[5], Hideto Imai[5], and Wataru Sugimoto[1,3,*]

[1] *Research Initiative for Supra-Materials (RISM), Shinshu University, 3-15-1 Tokida, Ueda, Nagano 386-8567, Japan*

[2] *Faculty of Science, University of the Ryukyus, 1-Senbaru, Nishihara, Nakagami, Okinawa 903-0213, Japan*

[3] *Faculty of Textile Science and Technology, Shinshu University, 3-15-1 Tokida, Ueda, Nagano 386-8567, Japan*

[4] *Office of Society-Academia Collaboration for Innovation, Kyoto University, Sakyo-ku, Kyoto 606-8501, Japan*

[5] *Device-functional analysis department, NISSAN ARC LTD., 1 Natsushima, Yokosuka, Kanagawa 237-0061, Japan*

*Corresponding authors:

daitaki@sci.u-ryukyu.ac.jp (D.T.), wsugi@shinshu-u.ac.jp (W.S.)

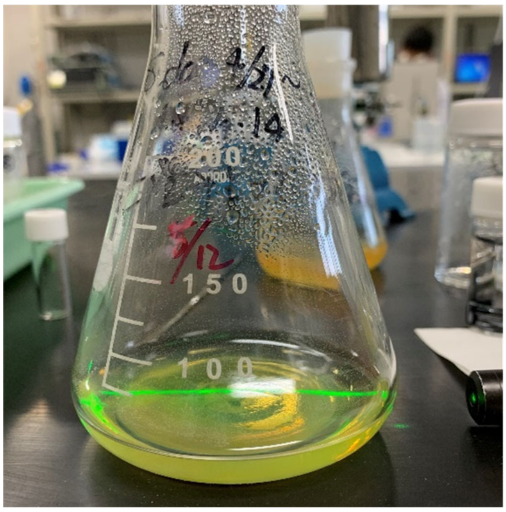

Supplementary Fig. 1 Photograph of a colloidal suspension of the PtO_x nanosheets (NSs) exfoliated from H_yPtO_x.

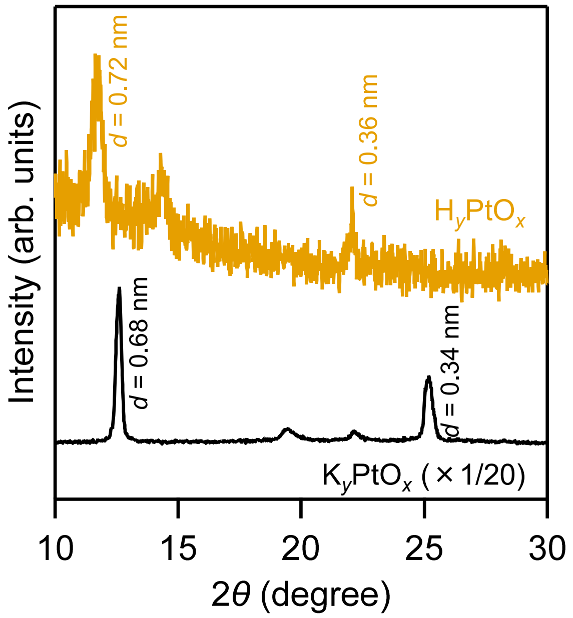

Supplementary Fig. 2 X-ray diffraction (XRD) patterns of K_yPtO_x and H_yPtO_x.

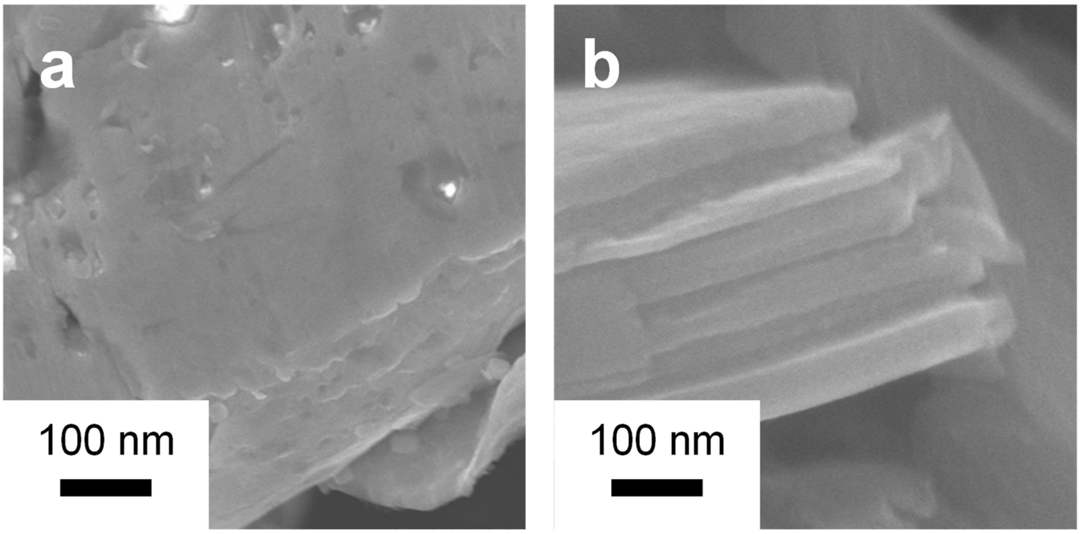

Supplementary Fig. 3 Typical scanning electron microscopy images of **a** K_yPtO_x and **b** H_yPtO_x.

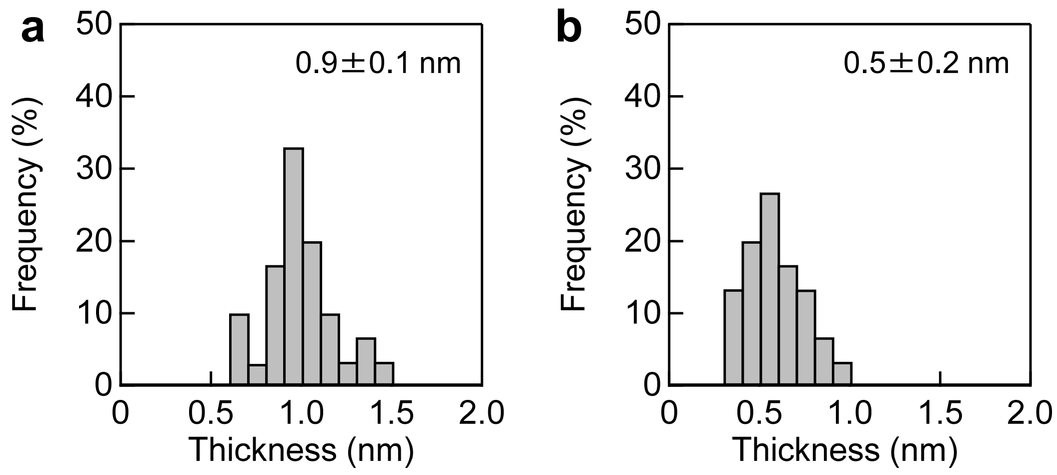

Supplementary Fig. 4 Thickness histograms of the **a** PtO_x (a total of 100 NSs) and **b** Pt NSs (total of 50 NSs).

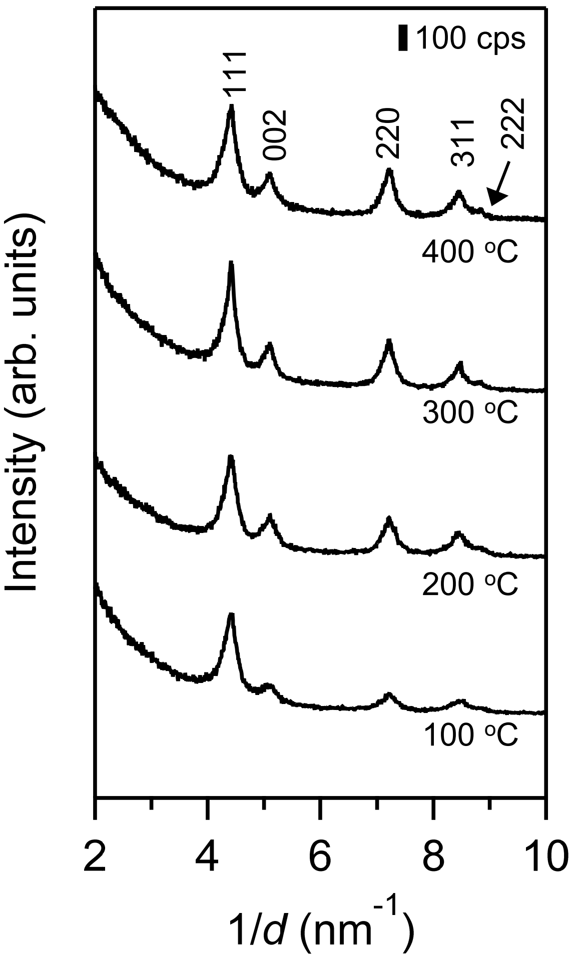

Supplementary Fig. 5 In-plane XRD patterns of PtO_x NS monolayer films heat-treated at 100, 200, 300, and 400 °C in H_2.

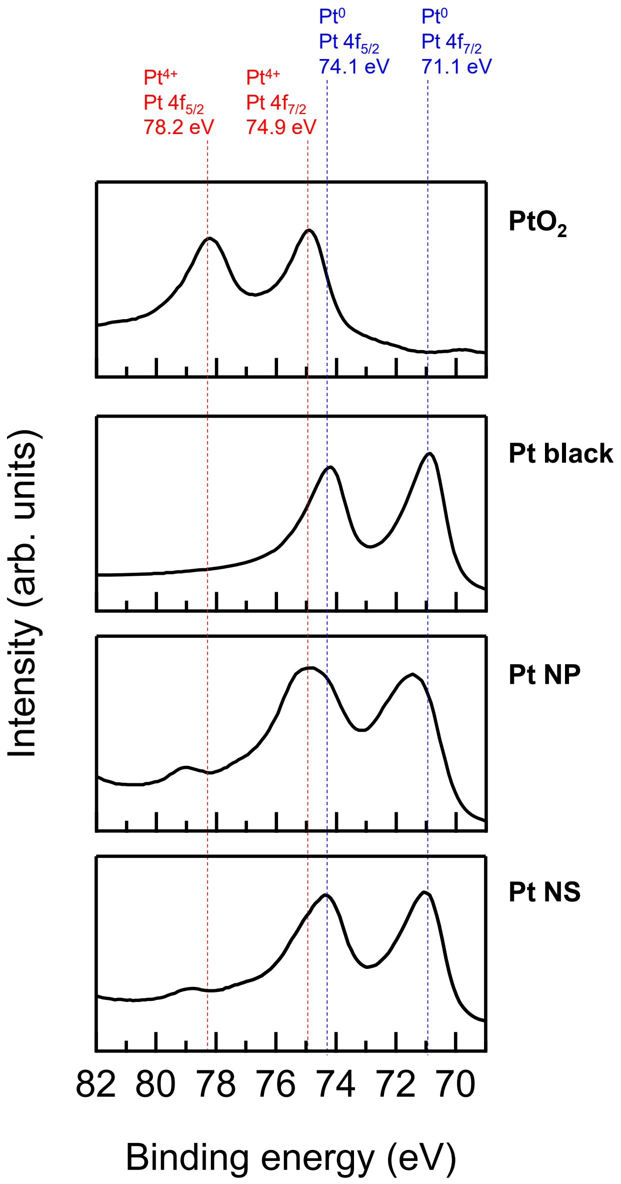

Supplementary Fig. 6 Typical X-ray photoelectron of PtO_2, Pt black, Pt nanoparticle (NP), and Pt NS.

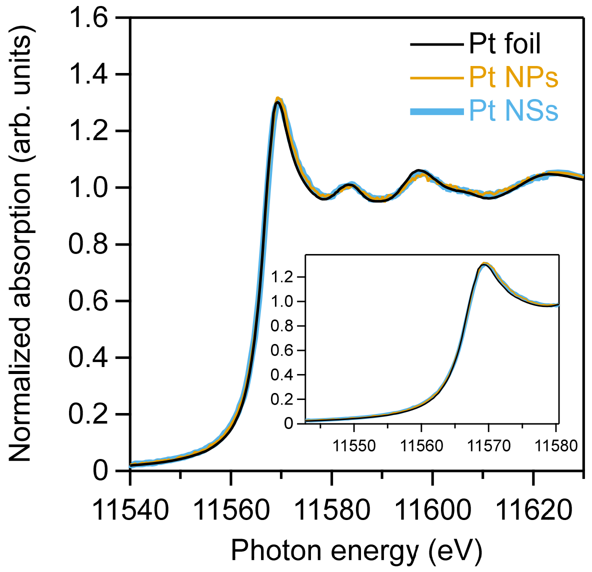

Supplementary Fig. 7 X-ray absorption near-edge structure spectra of the Pt foil, 3 nm-sized Pt NPs, and Pt NSs at 0.4 V vs. reversible hydrogen electrode (RHE).

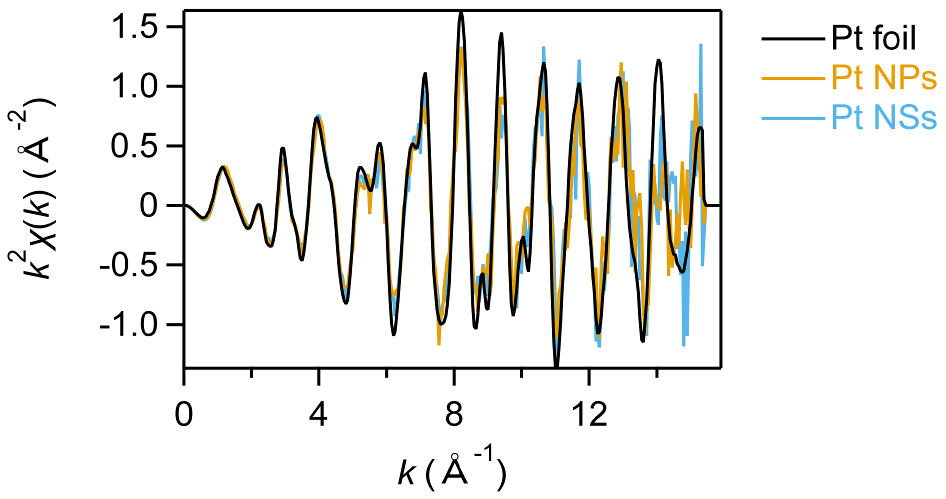

Supplementary Fig. 8 Spectra of Pt NSs with an oscillation period similar to that of Pt foil and 3 nm-sized Pt NPs.

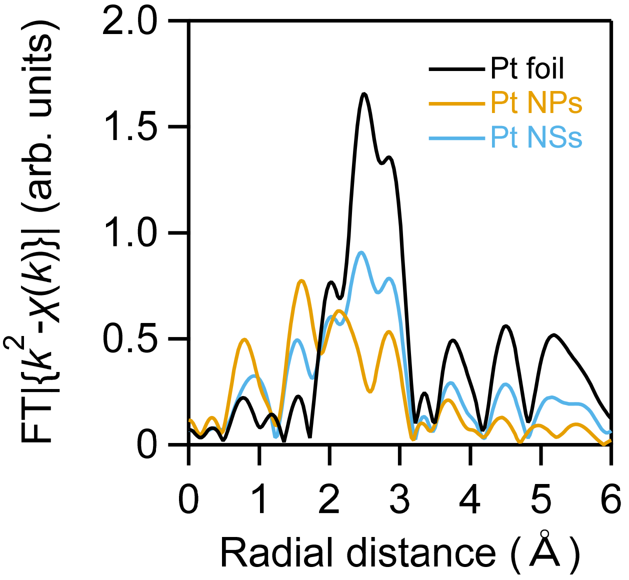

Supplementary Fig. 9 Fourier transform of the k^2-weighted extended X-ray absorption fine structure data of the Pt foil, 3 nm-sized Pt NPs, and Pt NSs at 0.4 V vs. RHE.

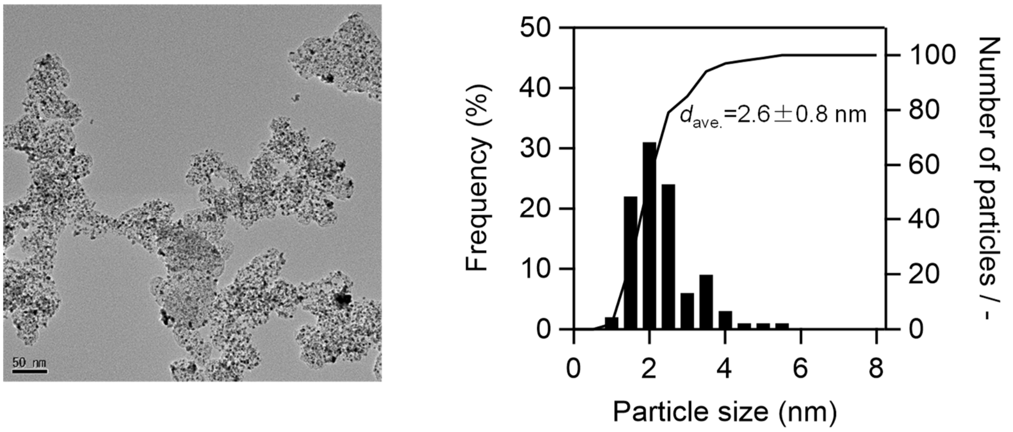

Supplementary Fig. 10 Typical transmission electron microscopy (TEM) images and corresponding particle size histograms of 3 nm-sized Pt NPs (TEC10E50E).

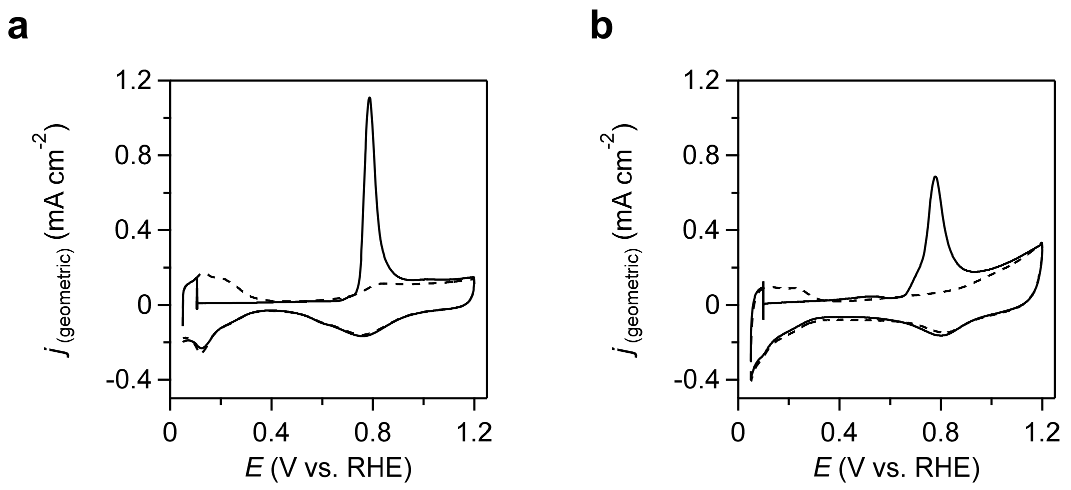

Supplementary Fig. 11 CO stripping voltammograms of **a** Pt NPs and **b** Pt NSs in 0.1 M $HClO_4$ (25 °C) at a scan rate of 10 mV s^{-1}. Broken line: the 1st cycle after CO adsorption; solid line: the 2nd cycle. The electrochemically active surface area (ECSA) values of Pt NSs and NPs are 160 and 100 m^2/g, respectively.

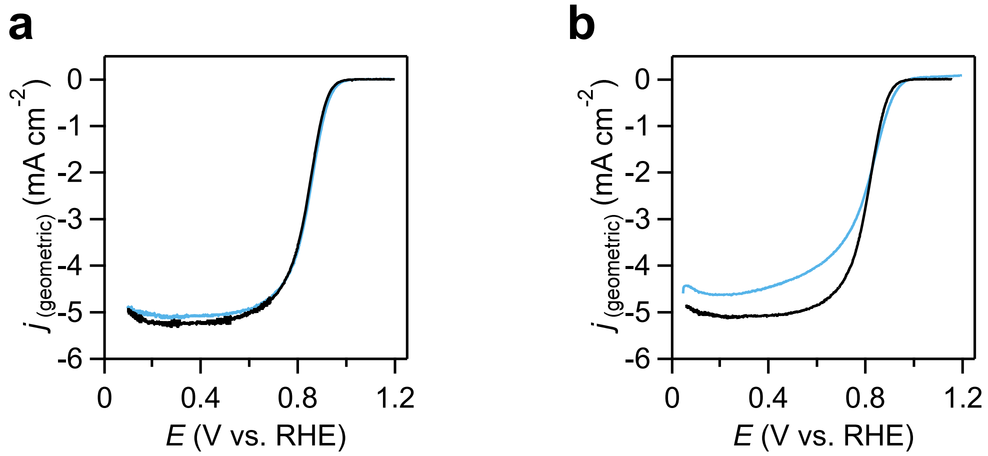

Supplementary Fig. 12 Linear sweep voltammograms **a** before and **b** after the durability test (0.6 (3 s)–1.0 (3 s) V vs. RHE at 60 °C; 5000 cycles) of Pt NPs (black) and Pt NSs (blue) in 0.1 M $HClO_4$ (25 °C) at v =10 mV s^{-1} and a rotation rate of 1600 rpm.

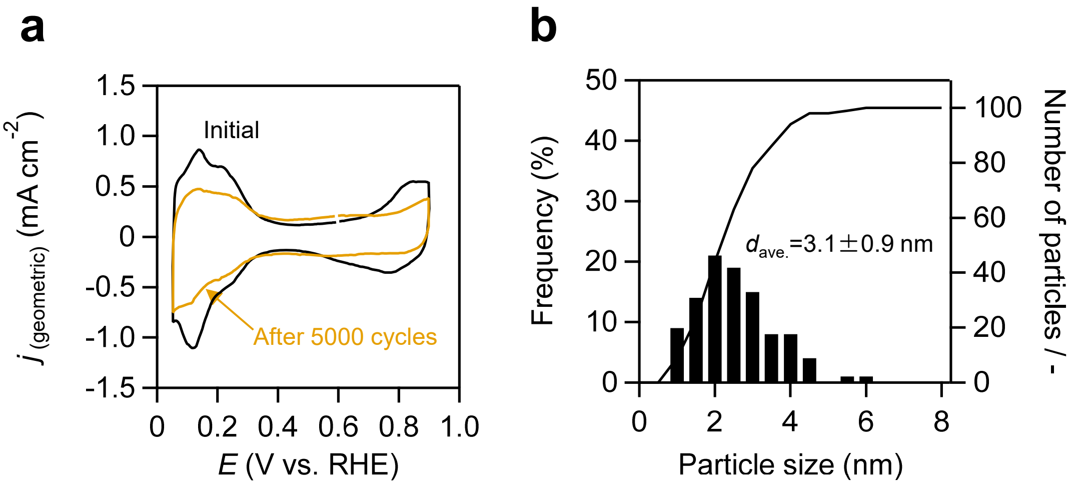

Supplementary Fig. 13 a Cyclic voltammograms of 3 nm-sized Pt NPs before and after 5000 cycles in 0.1 M $HClO_4$ (25 °C) at v =50 mV s^{-1}. **b** Pt-NP-size histograms of the 3 nm-sized Pt NPs after a durability test.

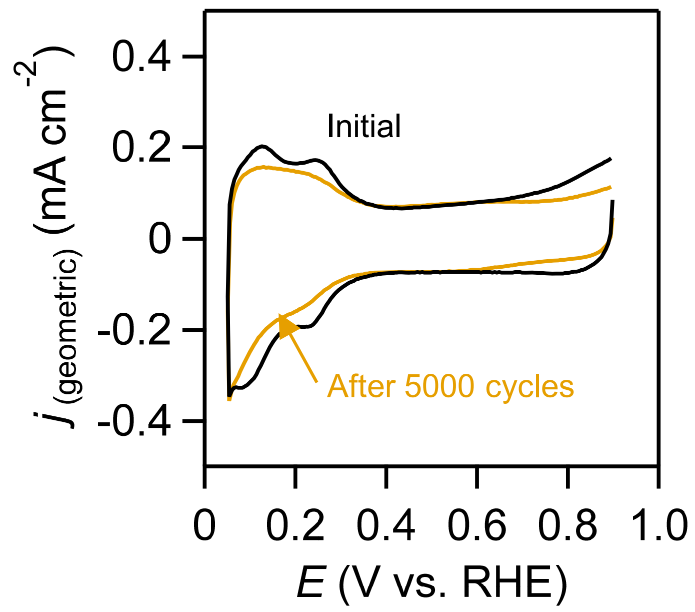

Supplementary Fig. 14 Cyclic voltammograms of Pt NSs before and after 5000 cycles in 0.1 M $HClO_4$ (25 °C) at v =50 mV s^{-1}.

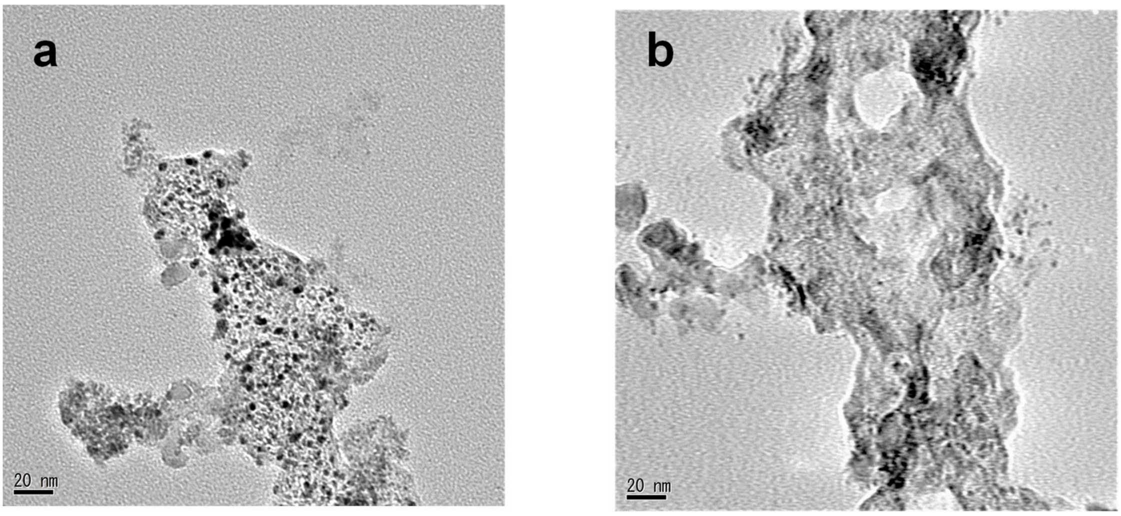

Supplementary Fig. 15 Typical TEM images after the durability test of **a** Pt NPs and **b** Pt NSs.

Supplementary Table 1 ECSA and oxygen reduction reaction (ORR) activity from Koutecky–Levich plots at 0.9 V vs. RHE of fresh samples.

	ECSA / m^2 g^{-1}	Specific activity / μA cm^{-2}	Mass activity /A g^{-1}
Pt NSs	124	330	415
Pt NPs	79	303	239

*Retention rates of the catalysts with respect to the initial mass activity are shown in parentheses.

Supplementary Table 2 ECSA and ORR activity from the mass-transport-included kinetic current at 0.9 V vs. RHE before and after the load-cycle accelerated durability test (ADT) test (0.6 (3 s)–1.0 (3 s) V vs. RHE at 60 °C; 5000 cycles)

	Initial			After ADT		
	ECSA / m^2 g^{-1}	Specific activity / μA cm^{-2}	Mass activity / A g^{-1}	ECSA* / m^2 g^{-1}	Specific activity* / μA cm^{-2}	Mass activity* / A g^{-1}
Pt NSs	124	333	413	93 (75%)	241 (72%)	224 (54%)
Pt NPs	79	295	233	45 (57%)	196 (66%)	74 (32%)

*Retention rates of the catalysts with respect to the initial values are shown in parentheses.
